# Brain Correlates of Single Trial Visual Evoked Potentials in Migraine: More Than Meets the Eye

**DOI:** 10.3389/fneur.2018.00393

**Published:** 2018-05-30

**Authors:** Marco Lisicki, Kevin D'Ostilio, Gianluca Coppola, Alain Maertens de Noordhout, Vincenzo Parisi, Jean Schoenen, Delphine Magis

**Affiliations:** ^1^Headache Research Unit, University of Liège, University Department of Neurology CHR Citadelle Hospital, Liège, Belgium; ^2^Research Unit of Neurophysiology of Vision and Neuro-Ophthalmology, G. B. Bietti Foundation IRCCS, Rome, Italy

**Keywords:** migraine, electrophysiology, single trial VEP, voxel-based morphometry, functional connectivity

## Abstract

**Background:** Using conventional visual evoked potentials (VEPs), migraine patients were found to be hyperresponsive to visual stimulus. Considering that a significant portion of neuronal activity is lost for analysis in the averaging process of conventional VEPs, in this study we investigated visual evoked responses of migraine patients and healthy volunteers using a different approach: single trial analysis. This method permits to preserve all stimulus-induced neuronal activations, whether they are synchronized or not. In addition, we used MRI voxel-based morphometry to search for cortical regions where gray matter volume correlated with single trial (st) VEP amplitude. Finally, using resting-state functional MRI, we explored the connectivity between these regions.

**Results:** stVEP amplitude was greater in episodic migraine patients than in healthy volunteers. Moreover, in migraine patients it correlated positively with gray matter volume of several brain areas likely involved in visual processing, mostly belonging to the ventral attention network. Finally, resting state functional connectivity corroborated the existence of functional interactions between these areas and helped delineating their directions.

**Conclusions:** st-VEPs appear to be a reliable measure of cerebral responsiveness to visual stimuli. Mean st-VEP amplitude is higher in episodic migraine patients compared to controls. Visual hyper-responsiveness in migraine involves several functionally-interconnected brain regions, suggesting that it is the result of a complex multi-regional process coupled to stimulus driven attention systems rather than a localized alteration.

## Introduction

**Figure 1:**
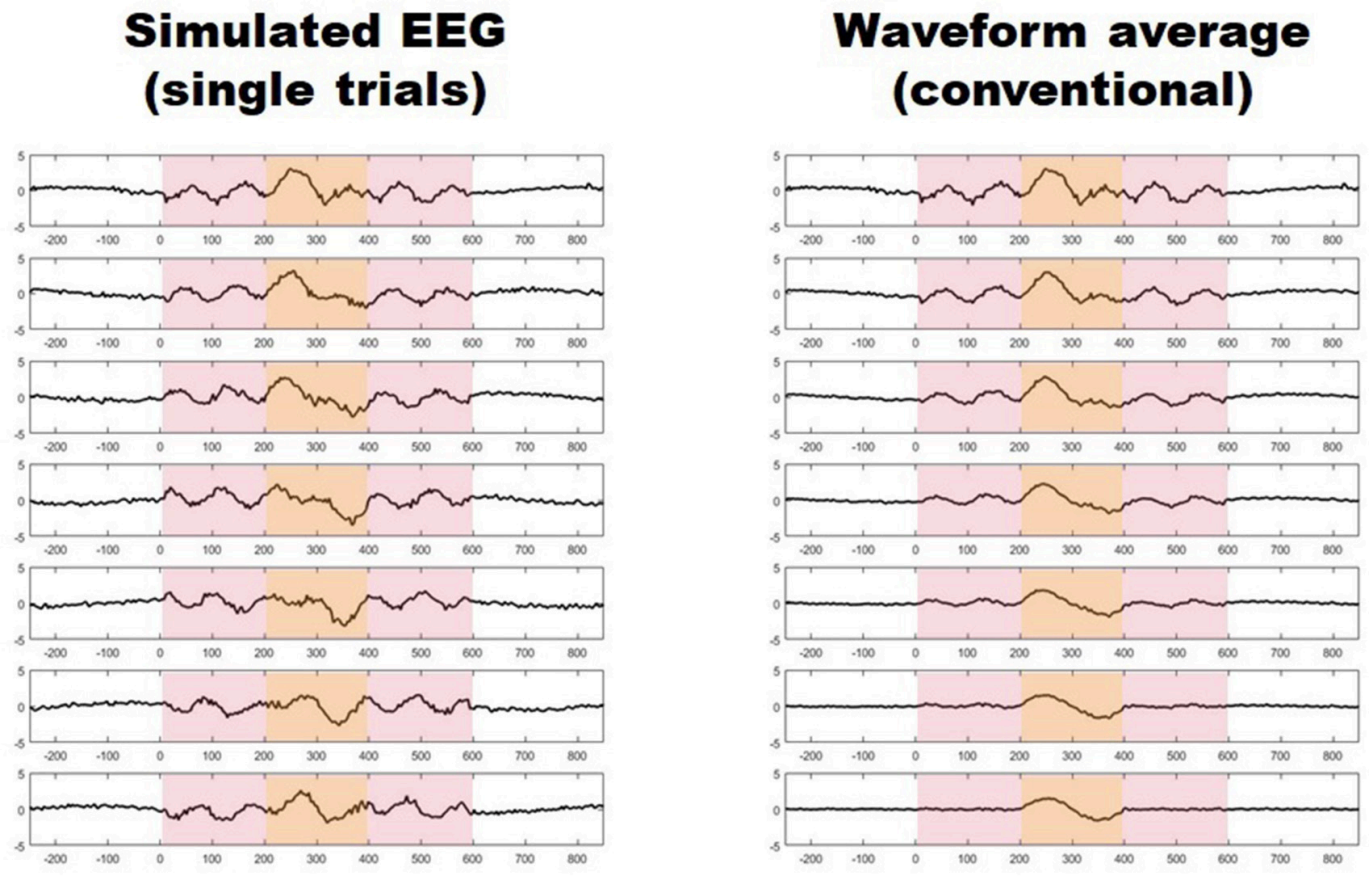
**(Left)** Seven simulated trials with stimulus induced activity occurring between 0 and 600 ms (light red shaded area). Only activity between 200 and 400 ms is synchronized (light yellow shaded area). **(Right)** Progressive waveform (point-by-point) average of the seven trials on the left, the paradigm of conventional evoked potentials. Notice how only synchronized activity withstands the averaging process while unsynchronized responses vanish after seven trials.

Migraine patients are thought to be hyperresponsive to visual stimuli (1). Despite major advancements (2), this phenomenon of which the underlying mechanisms remain largely undetermined, is often unseen by current methods (3). Using conventional transient visual evoked potentials, some investigators have found an increased amplitude in the responses of migraine patients with respect to healthy controls (4–9), while some others have not (10–12), and some have even found the initial amplitude to be relatively reduced (13, 14). Furthermore, asymmetric responses have also been reported in several studies (9, 15–17). These conventional visual evoked potentials (VEPs) are obtained by repeatedly presenting a visual stimulus and recording the electroencephalographic activity evoked in derivations over the visual cortex. In order to distinguish synchronized, stimulus-related, cortical activity from background noise (i.e., to increase the signal-to-noise ratio), hundreds of brief periods of post-stimulus electroencephalographic registry (trials), are point-by-point averaged. This method, however, may hide some important aspects of neuronal dynamics, since it may not capture unsynchronized, yet stimulus-induced, activity embedded in what is presumed to be noise (Figure 1) (18, 19). Indeed, analyzing all activity, both synchronized and unsynchronized, would be fundamental in order to obtain a truly comprehensive view of the neural processes that result from visual stimulation (18).Among the various methods of neurophysiological data analysis, single-trial analysis of evoked potentials (st-VEP) (i.e., analyzing trials one by one instead of averaging them into one single waveform) permits to measure all brain activity regardless of its synchronization, and may therefore provide a more comprehensive view of cortical stimulus-induced activation (20, 21).

Up to now, the sole study in which visual processing was addressed on a single-trial basis in migraine patients used steady state, instead of transient, VEPs (22), and reported an increased amplitude of the dominant frequency in patients with respect to controls, largely accounted for by unsynchronized EEG activity (22). To the best of our knowledge, transient pattern-reversal VEPs that have unraveled alterations of visual processing in migraine patients in numerous studies (23), have not yet been evaluated at the single trial level in the time domain, capturing all stimulus induced activity, and not only that occurring at specific frequencies (24). Furthermore, up till now VEP studies have not been combined with magnetic resonance imaging (MRI) in order to analyse the morpho-functional correlates of cortical hyper-responsiveness in interictal migraine.

In fact, although, several MRI studies have assessed structural integrity by means of voxel based morphometry (VBM) MRI (25) and interconnected networks with resting state functional connectivity MRI (fc-MRI) in the brain of migraine patients between and during attacks (26), the coexistence of functional and structural alterations was evaluated only in a few studies (27, 28) and, to the best of our knowledge, never for the visual system. By contrast, such correlative studies between VEPs and structure and function in visual areas have been performed in patients with optic neuritis (29, 30).

The aim of the present study was to analyse transient pattern reversal VEPs on a single trial basis (st-VEP) in episodic migraine patients and, for comparison, in healthy volunteers and to search for correlations between mean st-VEP amplitude and structural brain changes using VBM MRI. In a second step we analyzed connectivity between those clusters of gray matter that were correlated with st-VEP using resting state functional MRI (fc-MRI).

## Subjects and methods

### Subjects

The study involved 40 right-handed subjects: 20 healthy volunteers (HV, mean age: 34.8 ± 11.3 years, 15 female/five male), and 20 episodic migraine without aura patients (EM, mean age: 32.2 ± 12.8 years, 16 female/four male) diagnosed in accordance to The International Classification of Headache Disorders 3rd edition (Beta version) (31). Participants were recruited among University students or their families or via our headache clinic. Most females were under hormonal contraceptive regimes. Subjects undergoing any other medical treatment were not allowed to participate. Specifically, EM patients were not under any migraine preventive treatment at the time of recordings, and for at least 90 days preceding them. All participants were free of any systemic or neurological disease other than migraine. Healthy volunteers did not report any first degree relative suffering from recurrent headache of any type. The mean number of monthly migraine days determined with a headache diary was 4.1 ± 2.6. The patients' headaches were not side-locked. Migraine patients were recorded at an interval of at least 72 h after and 72 h before an attack verified by diary inspection. The study was approved by the Institution's ethics committee (Centre Hospitalier Régional de la Citadelle, Liège, Belgium—protocol n°1422) and conducted following the principles of the Declaration of Helsinki. All participants gave their written informed consent.

## VEP recordings and analysis

### VEP acquisition

VEP recordings were performed in the electrophysiology laboratory of our Headache Research Unit (Neurology Department, Centre Hospitalier Régional de la Citadelle, Liège, Belgium). Subjects sat on a comfortable armchair, in a quiet room with dimmed light. They were instructed to spot a red dot in the center of a screen displaying a black and white checkerboard pattern (contrast of 80%, mean luminance 50 cd/m2) at temporal and spatial stimulating frequencies of 1.55 Hz (3.1 reversals/s) and 68° respectively. Needle recording electrodes were placed at Oz (active) and Fz (reference) of the 10–20 EEG system. Six hundred epochs were continuously recorded using a pattern reversal monocular stimulation with the left eye patched. Signals were recorded using a CED^TM^ power 1401 device (Cambridge Electronic Design Ltd, Cambridge, UK). Consecutive sweeps, each lasting 250 ms, were collected at a sampling rate of 5.000 Hz using the Signal software package version 4.02 (Cambridge Electronic Design Ltd, Cambridge, UK). Investigators performing the VEP recordings were not blinded to patients' diagnosis. After DC subtraction, recordings were exported to EEGLAB (20), an open-source MATLAB (The MathWorks Inc.) toolbox for electrophysiological signal processing, where they were band-pass filtered (low pass 100 Hz, high pass 1 Hz). Afterwards, artifacted epochs exceeding a two standard deviations of the channel mean limit were rejected (<7% of epochs).

### Single trial analyses

**Figure 2:**
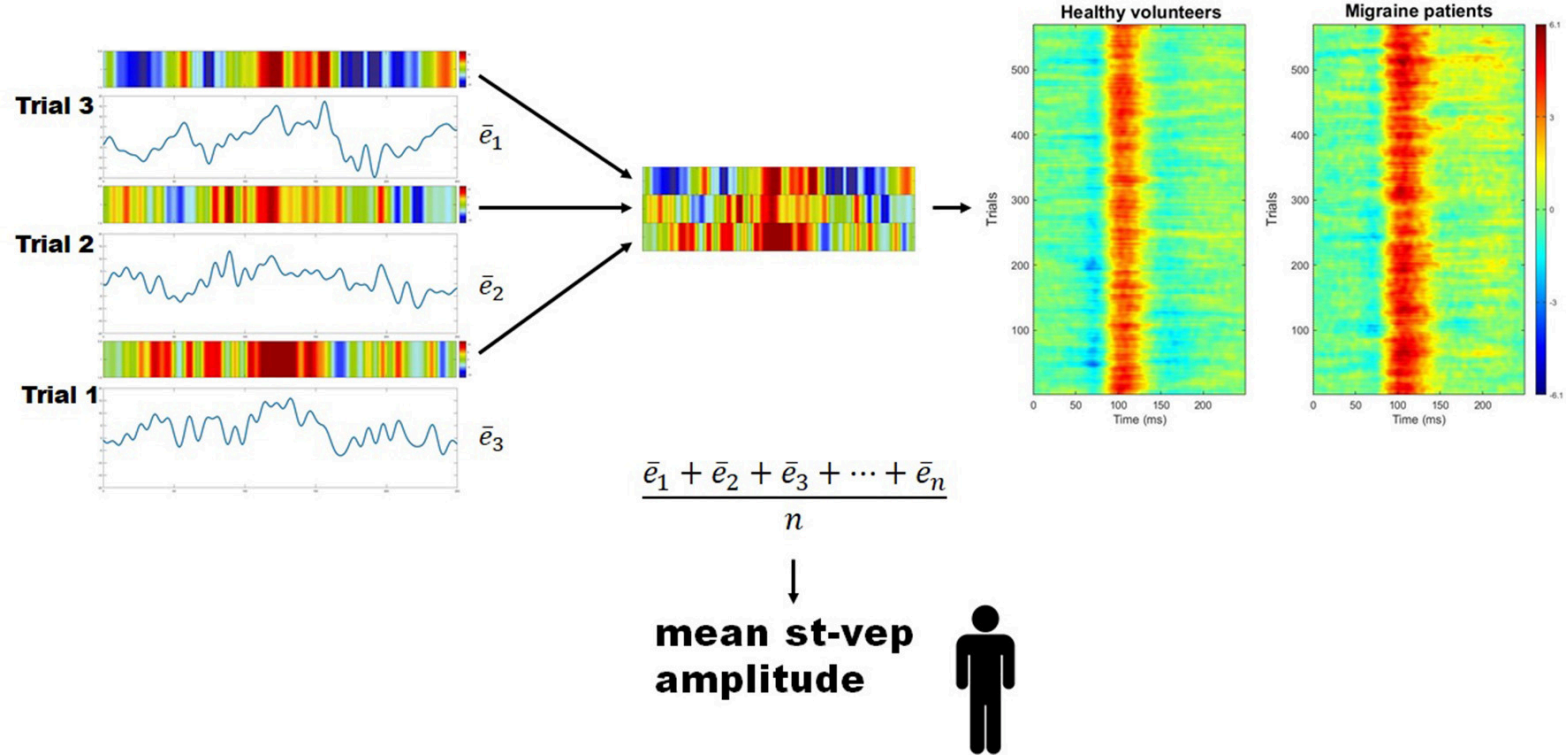
Mean single trial visual evoked potential (st-VEP) amplitude. Three trials are taken as example (left). For each trial, mean amplitude throughout the epoch is calculated ($\bar{e}$). The sum of these means is then divided by the total number of un-artifacted trials in order to obtain the averaged mean single trial visual evoked potential (st-VEP) amplitude of each participant. Contrary to waveform average, this allows to preserve all stimulus induced activity, regardless of phase synchronization. For visual inspection, each trial is color-coded (colored bars over the electric traces). Colored representations of trials are subsequently stacked together (detail, center), resulting in images similar to those on the right after putting together a considerable amount of trials. Thereafter, the presence of a response, unnoticeable on isolated trials, becomes evident. Here, group results are plotted for displaying purposes. Notice the increased response in migraine patients at approximately 100 ms (red area).

For every subject, the mean amplitude (voltage) throughout the whole duration of each individual trial (epoch) was calculated ($\bar{e}$) using a EEGLAB toolbox (Figure 2). These values were summed, and the total was divided by the number of trials. By doing so, a grand average mean st-VEP amplitude was obtained for each participant (see Figure 2).

### Conventional VEP analyses

For conventional VEP analyses, an averaged waveform was generated by point-by-point averaging all trials of each patient together. This resulted in the classic VEP wave. The N1 peak was defined as the most negative point between 60 and 90 ms after the stimulus. P1 was defined as the most positive point following N1 between 80 and 120 ms. N1-P1 peak-to-peak amplitudes were measured and compared between groups.

## Statistical analysis of VEP measures

Statistical analyses and graphs were performed in Prism version 6.00 for Windows (GraphPad Software, La Jolla, California, USA). The assumption of normality was tested using a Shapiro-Wilk normality test. All continuous variables followed a normal distribution. Mean amplitudes were compared with Student's *t*-test. The significance level was set at *p* < 0.05.

## MRI acquisitions and analysis

### MRI acquisitions

On a separate day with respect to VEP recordings (median elapsed time: 11 days; interquartile range: 14.5), patients underwent 3T BOLD resting-state functional MRI [Siemens Allegra scanner (Siemens AG, Munich, Germany)] in the Radiodiagnostics Department of the Centre Hospitalier Universitaire de Liège (Pr R. Hustinx, Dr L. Tshibanda). Three hundred multislice T2^*^-weighted fMRI images were obtained with a gradient echo-planar sequence using axial slice orientation (32 slices; repetition time = 2000 ms, echo time = 30 ms, field of view = 240 mm, voxel size = 3.75 × 3.75 × 3.6 mm^3^; matrix size 64 × 64 × 36; flip angle = 90°; field of view = 240 mm). In addition, T1 structural magnetic resonance images were acquired (TR = 2300 ms, TE = 2.47 ms, T1-weighted 3D-gradient echo images with 1 × 1 × 1.2 mm^3^ voxels in the sagittal plane, flip angle = 9°, matrix size = 256 × 240 × 144 mm^3^, field of view = 256 mm).

### Voxel based morphometry (MRI)

MRI data were processed using the Statistical Parametric Mapping software (SPM 12, Wellcome Trust Centre for Neuroimaging, London, UK; http://www.fil.ion.ucl.ac.uk/spm) implemented in MATLAB 16 (Mathworks Inc., Sherbom, MA).

T1 structural images were first reoriented before pre-processing. After segmentation into white matter, gray matter, and CSF, normalization was performed using DARTEL to allow for high-dimensional spatial normalization. After VBM pre-processing, resulting gray and white matter segments were smoothed with a 6 mm kernel. The smoothed images were used for statistical analysis.

Thereafter, we performed a univariate regression analysis of gray matter volume including mean st-VEP amplitude as regressor. Analyses were performed controlling for whole brain size. Identification of cerebral areas and masks were obtained with the WFU PickAtlas toolbox (Wake Forest University School of Medicine, Advanced NeuroScience Imaging Research lab (ANSIR), Winston-Salem, NC, U.S.A) (32). Statistical significance was set at *p* < 0.001 uncorrected.

### Data driven exploratory resting state connectivity analyses: functional MRI (fMRI)

Based on the primary results, specific volumetric regions of interest (ROI) were generated using the clusters where st-VEP showed a positive correlation with gray matter volume in episodic migraine patients. In order to assess functional connectivity between these cerebral regions, we performed ROI-to-ROI connectivity analyses with the seed placed in the cluster displaying the highest st-VEP amplitude-to-gray matter volume correlation.

In each session, the first three volumes were discarded to allow for T1 saturation effects. Data were pre-processed and analyzed using SPM12 (Wellcome Trust Centre for Neuroimaging, http://www.fil.ion.ucl.ac.uk/spm) implemented in MATLAB 16 (Mathworks Inc., Sherbom, MA) and CONN connectivity toolbox (The Gabrieli Lab. McGovern Institute for Brain Research, Massachusetts Institute of Technology, U.S.A) (33). For each subject, we first applied a slice-timing correction to compensate for the staggered order of slices acquired. The time series were then spatially realigned using rigid body transformations that minimize the residual sum of square between the first and each subsequent image. The mean image created from the realigned time-series was spatially co-registered to the anatomical MRI image and co-registration parameters were applied to the realigned time series. Normalization parameters were subsequently applied to the co-registered times series, then re-sliced to a voxel size of 2 × 2 × 2 mm^3^, and spatially smoothed using an 8 mm full width at half maximum Gaussian kernel. A noise correction and a temporal band-pass filter of 0.008–0.09 Hz were applied on the time series to restrict the analysis to low frequency fluctuations, which characterize functional blood-oxygen-level dependent contrast imaging resting state activity.

Because of technical problems (e.g., artifact, difference in number of slices, head movements) rs-fMRIs of one subject could not be included in the analyses.

Volumetric masks of clusters of gray matter containing voxels positively correlated to mean st-VEP amplitude above a peak T = 4.0 threshold were included as seeds/sources. The 14 ROIs included are detailed in Table 1. For connectivity analysis, the seed was placed in the ROI derived from the cluster with the highest statistical level of correlation with the st-VEP amplitude [right temporo-parietal junction (rTPJ), T = 7.07 in VBM analysis.

**Table 1:** Gray matter correlates of single trial visual evoked potentials.

			**Cluster**				**Peak**	**Peak**	**peak**
			**Voxels**	**x,y,z {mm}**	**x,y,z {mm}**	**x,y,z {mm}**	**p(unc)**	***T***	**equivZ**
**MIGRAINE PATIENTS**									
1	seed	Right TPJ (infero-medial)*	112	42	−57	28.5	0.0000007	7,06682014	4,82969372
2	·	Left TPJ (superior temporal gyrus)	265	−42	−52.5	13.5	0.0000189	5,41838455	4,12002826
3	·	Right calcarine cortex	138	15	−78	7.5	0.0001098	4,60539389	3,695312
4		Right cerebellum	73	45	−64.5	−55.5	0.0001114	4,59864664	3,69154176
5	·	Right TPJ (supero-lateral)	56	52.5	−57	49.5	0.0001560	4,44605017	3,60512258
				45	−57	54	0.0005979	3,84129429	3,23989488
				57	−57	40.5	0.0009405	3,63785815	3,10841684
6	·	Left cerebellum	171	−43.5	−61.5	−57	0.0001711	4,40432739	3,58110512
				−42	−76.5	−46.5	0.0002026	4,32794714	3,53669723
7		Right superior occipital gyrus	52	22.5	−79.5	45	0.0001832	4,37347031	3,56323348
8	·	Left post-central gyrus	32	−12	−37.5	76.5	0.0002073	4,31763268	3,53065637
9	·	Right middle frontal gyrus	298	27	30	42	0.0002677	4,20224047	3,4623527
				28.5	24	55.5	0.0004553	3,96353984	3,31676094
10	·	Right middle temporal gyrus	47	69	−46.5	−4.5	0.0003112	4,13454056	3,42165515
11	·	Left middle frontal gyrus	38	−28.5	30	49.5	0.0003691	4,05785036	3,37498704
12		Left cuneus	51	−18	−70.5	28.5	0.0003837	4,04041004	3,36428942
13	·	Left calcarine cortex	29	−15	−76.5	7.5	0.0004090	4,01174641	3,34663897
14		Left middle frontal gyrus	73	−31.5	30	34.5	0.0004110	4,00951338	3,34526033
**HEALTHY CONTROLS**									
1		Right TPJ	51	42	−55.5	27	0.0000705	4,80743933	3,80626101
2		Left superior frontal gyrus	5	−13.5	24	63	0.0004295	3,98979211	3,33306205
3		Left superior frontal gyrus	2	−7.5	40.5	54	0.0006120	3,83077836	3,23320829
4		Right superior frontal gyrus	3	9	1.5	72	0.0006326	3,81591582	3,22373767
5		Right superior frontal gyrus	5	10.5	6	70.5	0.0007351	3,7485621	3,18052061
6		Left superior frontal gyrus	3	−7.5	37.5	55.5	0.0009264	3,64462614	3,11286349

**Figure 3:**
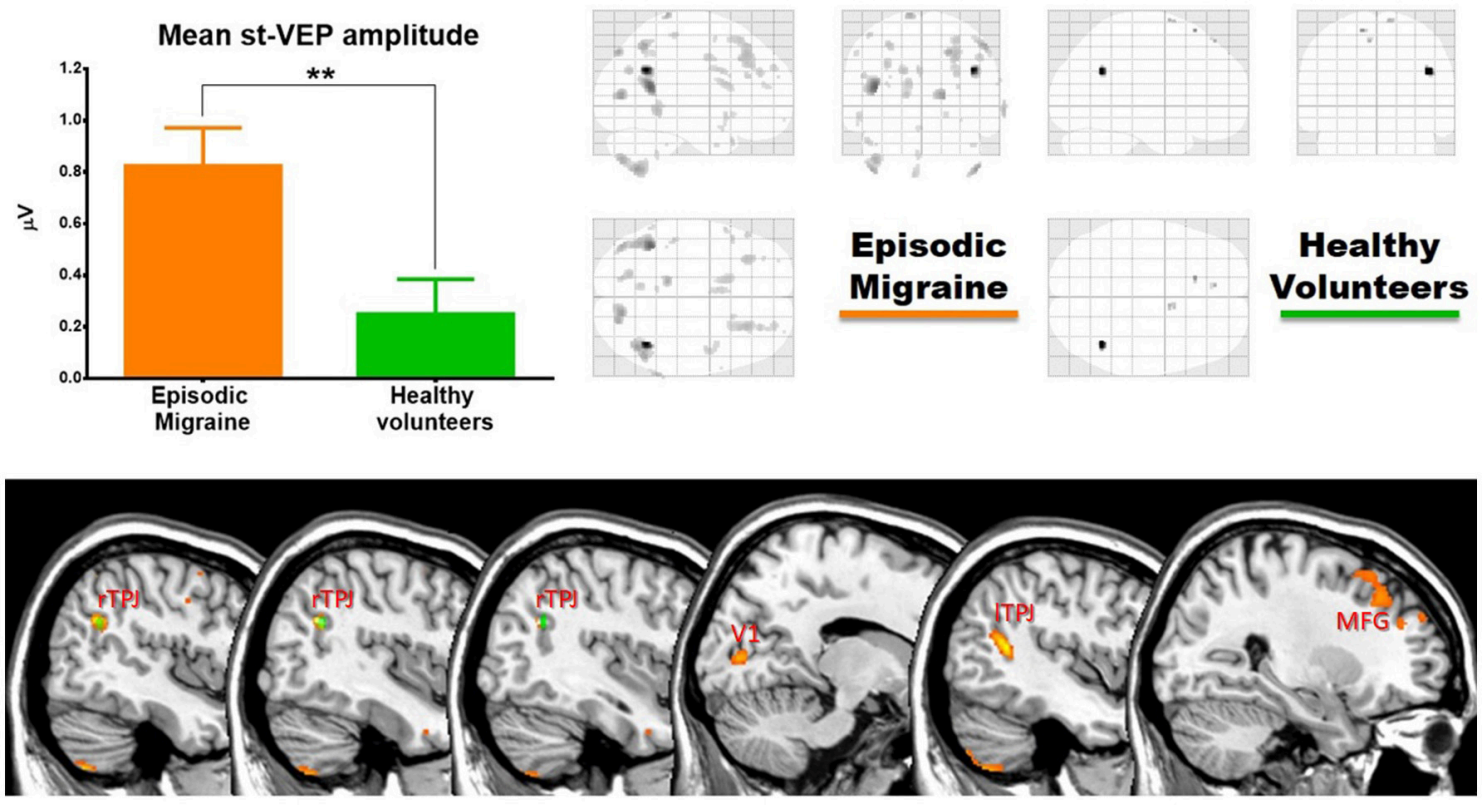
**(Top)** Left, bar graph of amplitudes of single trial visual evoked potentials. Error bars indicate the standard error of the mean. **p < 0.01. Right, voxels of gray matter volume positively correlated with mean single trial visual evoked potential amplitude in sagittal, coronal, and axial planes of a “glass brain” (*p* < 0.001 uncorrected). **(Bottom)** The same regions projected over the sagittal slices of a T1 template (from left to right x = 45, 42, 40, 14, −44, and 27). First three slices show voxels positively correlated with mean st-VEP amplitude in migraine patients (orange-yellow) and healthy volunteers (green) within the right (rTPJ) temporo-parietal junction. Subsequent slices show other cortical regions [visual cortex (V1), left the temporo-parietal junction, and middle frontal gyrus (MFG) respectively] exhibiting correlations only in migraine patients.

Clusters of gray matter containing voxels positively correlated to mean st-VEP amplitude above a peak T = 4.0 threshold in episodic migraine patients and healthy controls. All clusters contained voxels correlated with mean st-VEP amplitude at p < 0.001 uncorrected. *p family-wise error [FWE]-corr < 0.05 at whole brain level. The 14 ROI included as seeds/sources for connectivity analysis in episodic migraineurs were generated using volumetric masks of the clusters in this table. ROI displaying significant [p false discovery rate (FDR) < 0.05] resting state functional connectivity with the seed are marked with a dot (·) in the second column. For details of connectivity analyses see Table 2, Figure 3. TPJ, temporo-parietal junction.

## Results

### Visual evoked potentials

Single-trial transient pattern-reversal VEP amplitude was significantly greater in EM patients (0.824 μV ± 0.661) than in healthy controls (0.250 μV ± 0.605) (*p* = 0.007) (Figure 2). By contrast, the N1-P1 amplitude of the conventional VEP did not differ significantly between groups (HV = 5.895 μV ± 1.509, EM = 6.201 μV ± 1.757; *p* = 0.56).

### Voxel-based morphometry

In EM patients, mean st-VEP amplitude was positively correlated with gray matter volume in the right temporo-parietal junction (rTPJ), peaking at the angular gyrus [whole brain analysis: 42, −57, 29 p family-wise error (FWE) = 0.006 T = 7.07]. Additionally, the bilateral primary visual cortex, the left temporo-parietal junction, bilateral middle frontal gyrus, right superior occipital gyrus, right inferior parietal cortex, left post-central gyrus, left cuneus, and bilateral cerebellum, showed positive correlations with st-VEP amplitude at an uncorrected *p* < 0.001 threshold level (Table 1, Figure 3).

In healthy volunteers, the observed correlation with the right angular gyrus was weaker than in patients (whole brain analysis: 42, −56, 27, *p* < 0.001 uncorrected threshold T = 4.81), and only small (≤5 voxels) clusters were observed in the superior frontal gyri. There was no correlation between mean st-VEP amplitude and gray matter volume in the visual cortex.

Controlling for whole brain size, no overall differences in gray matter volume were observed between the groups.

### Data driven exploratory connectivity analyses (fMRI)

**Figure 4:**
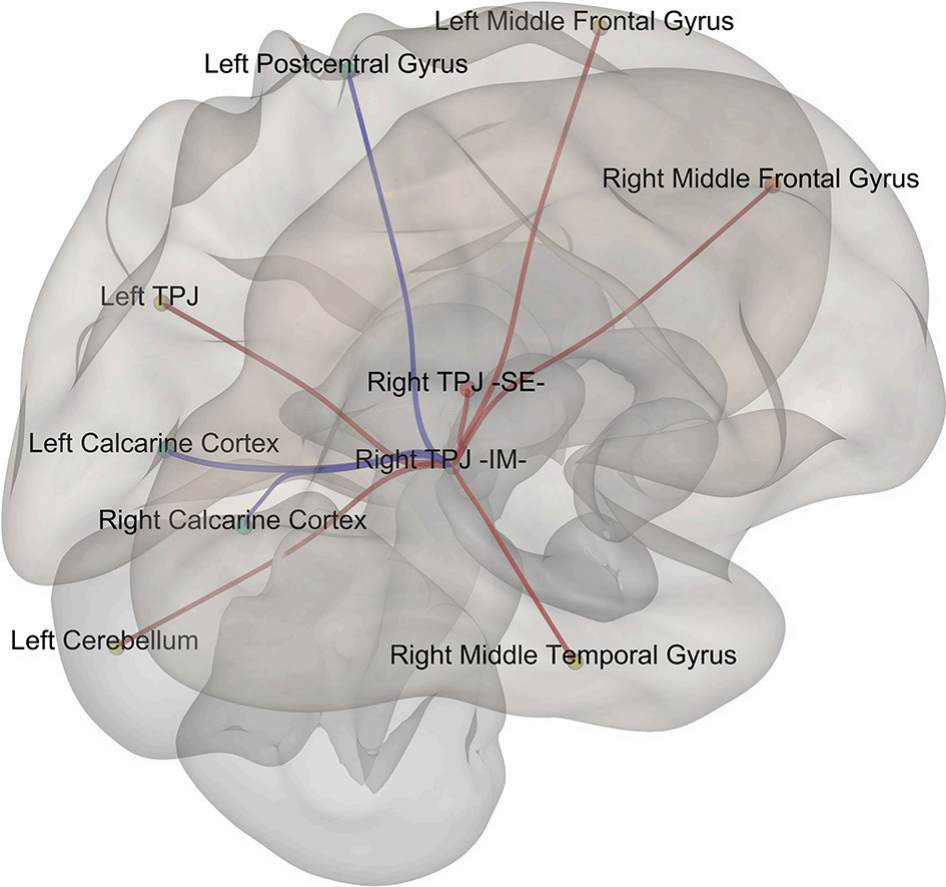
Episodic Migraine Patients' resting-state fMRI connectome seeded in the right temporo-parietal junction (infero-medial). ROI were generated using the clusters of gray matter volume positively correlated with mean st-VEP amplitude (detailed in Table 1). Only significant (p-FDR < 0.05 -seed level correction) connections are shown in the figure. Positive interactions are depicted in red and negative interactions are depicted in blue. For details see Table 2.

Given that it exhibited the stronger correlation with mean st-VEP amplitude in terms of gray matter volume, functional connectivity analyses were seeded in the right temporo-parietal junction. Significant [*p*-false discovery rate (FDR) < 0.05] interactions between this marked region and nine of the other derived ROIs were found. Negative interactions were observed between the seed and the left post-central gyrus as well as the calcarine cortices; conversely, interactions with all the other ROIs were positive (Table 2, Figure 4).

**Table 2:** Connectivity in episodic migraine patients.

**Seed**	**Analysis unit**	**Statistic**	**p-FDR**
Right TPJ -IM-	Right TPJ -SE-	*T*_(18)_ = 8.24	0.0000
Right TPJ -IM-	Right middle frontal gyrus	*T*_(18)_ = 7.04	0.0000
Right TPJ -IM-	Left middle frontal gyrus	*T*_(18)_ = 4.10	0.0029
Right TPJ -IM-	Left cerebellum	*T*_(18)_ = 3.63	0.0062
Right TPJ -IM-	Left TPJ	*T*_(18)_ = 3.49	0.0068
Right TPJ -IM-	Left calcarine cortex	*T*_(18)_ = −3.21	0.0104
Right TPJ -IM-	Right middle temporal gyrus	*T*_(18)_ = 3.14	0.0104
Right TPJ -IM-	Right calcarine cortex	*T*_(18)_ = −2.83	0.0180
Right TPJ -IM-	Left poscentral gyrus	*T*_(18)_ = −2.39	0.0405

*Resting state functional connectivity analysis between clusters were gray matter is correlated with single trial visual evoked potentials' amplitude in episodic migraine patients (as schematized in Figure 3). Only ROI exhibiting significant p-FDR < 0.05 connections are included. TPJ, temporo-parietal junction; IM, infero-medial*.

## Discussion

This is the first study analyzing transient pattern-reversal VEPs with a single trial method (st-VEP) in episodic migraine patients. We found that neural activation to single visual stimulus, as reflected by st-VEP, is greater in episodic migraine patients than in healthy subjects. When we searched with MRI for the anatomo-functional correlate of this electrophysiological measure in terms of gray matter volume and functional connectivity, we found that mean st-VEP amplitude was proportional to gray matter volume in the visual cortex as in cerebral areas belonging to the ventral attention network (34). Indeed, the hub of this network, the right temporo-parietal junction (rTPJ), showed the strongest correlation regarding gray matter volume, and displayed significant functional connections with most of the other st-VEP correlated brain regions. Taken together, these results suggest that cerebral responsiveness to visual stimuli, as assessed with st-VEP, is exaggerated in interictal migraine, and correlated with gray matter volume, not only in the visual cortex, but also in a series of functionally connected brain areas involved in attention control.

Unlike for conventional VEPs that are affected by both synchronization and amplitude modifications, the only determinant of st-VEP is the amplitude of the cerebral response. This is an advantage when looking for a quantitative measurement of brain responsiveness, not biased by other parameters of neural dynamics. In nature, brain responses occur in “single trials.” Neurophysiologists use to point-by-point average event-related potentials based on the misconception that unsynchronized activity represents “noise” in terms of sensory processing. Even though the conventional approach allows obtaining interesting information and remains advantageous for some purposes, it is not otpimally suited for raw estimations of cortical responsiveness. As illustrated in our study, single trial VEP analysis provides a more global measure of sensory activation granting, contrary to the conventional averaging techniques, a comprehensive assessment of the role of visual responsiveness in migraine pathophysiology.

In our study, migraine patients had a significant correlation between st-VEP amplitude and gray matter volume in several brain regions, including the visual cortex and areas of the ventral attention network. The dorsal and ventral attention networks of the brain function interactively to direct attention toward specific targets (34). They broadly comprise the inferior parietal sulcus and frontal eye field for the dorsal network, and the right temporo-parietal junction and ventral frontal cortex for the ventral network (35). The ventral attention network shows a high degree of laterality to the right (34). From a functional perspective, while the dorsal attention network directs attention in a top–down manner (cognitively directed), the ventral attentional network is in charge of quickly recognizing salient, biologically relevant stimuli and redirecting attention toward them (i.e., bottom-up). These selective attention processes are of crucial importance as they underlie fundamental adaptive behaviors.

The involvement of the temporo-parietal junction in migraine pathophysiology was suggested by several studies. Using ^15^O-H_2_O PET to measure cerebral blood flow, Weiller et al., observed significant increases bilaterally in the temporo-parietal junction (BA 39/19) during spontaneous migraine attacks with respect to the headache-free interval (36). In one report pulsating headache in migraine was suggested be correlated to neuronal activity in parieto-occipital regions of the cortex rather than to arterial pulsations (37). More recently, in a low resolution electromagnetic tomography (LORETA) study, Clemens, et al. found that alpha activity was increased in this cortical area in migraine patients compared to controls (38). In addition, based on their findings in behavioral experiments (39), Mickleborough et al. designed an fMRI study to assess attentional control networks during visual spatial-orienting tasks in migraine patients and controls. Although the two groups showed activation in the key areas of attentional processing networks, migraineurs exhibited less activation than controls in the right temporo-parietal junction (40). Based on the results of both studies, the authors conclude that migraine patients lack attentional suppression of unattended events and have heightened orienting to sudden onset stimuli in the environment (40). Another research group, using a stimulation pattern designed to strongly activate the visual cortex (rather than to selectively evaluate attention), found increased activation of the temporo-parietal junction of the affected hemisphere in migraine patients with side-locked aura in comparison with the contralateral hemisphere or to healthy volunteers (41). Mainero et al., reported increased functional resting-state connectivity between the right supramarginal gyrus and the periaqueductal gray (PAG) that correlated positively with migraine attack frequency (42). In a combined resting state-fMRI study of the whole brain with diffusion tensor imaging (DTI), evidence for abnormal connectivity between the dorsal and ventral attention networks and the executive control network as well as the thalamus, were observed in migraine during attacks (43). There is thus convergent, though still fragmentary, evidence that the temporo-parietal junction deserves attention in future studies of migraine pathophysiology.

Because of the negative functional connectivity between the rTPJ and the striate cortex found in our study, one may hypothesize that a reduced inhibition of the visual cortex by the rTPJ might in part be responsible for increased st-VEP amplitudes found in EM patients (i.e., migraineurs seem unable to silence behaviorally irrelevant visual stimuli). Such a gatekeeper function for the TPJ has been suggested for other sensory modalities like olfaction, where reduced activation (or even de-activation) of the TPJ was associated with increased activation of the primary olfactory cortex (44). Indeed, the ability of the ventral attentional network to identify and direct attention to a relevant object in the environment involves other sensory modalities besides the visual (45).

Gray matter volume changes may be depend on neuronal or glial cell bodies, spine density and synapse plasticity, regional blood flow and interstitial fluid, all of which are directly or indirectly related to neuronal activity (46, 47). In this study we failed to find baseline differences in gray matter volume between healthy volunteers and migraine patients. Mean st-VEP amplitude in EM patients was nonetheless positively correlated with gray matter volume in the visual cortex (BA 17) as well as in other areas discussed above. From our cross-sectional study we cannot determine whether the higher functional activation of the visual areas and ventral visual attention network drives the morphological changes or vice-versa. However, since st-VEP amplitude, but not gray matter volume, was significantly greater in EM than in healthy subjects, it seems likely that the heightened functional activation results in plastic tissue changes in the visual areas that are too subtle to be detectable on group comparisons of VBM MRI.

Besides their contribution to the understanding of migraine pathophysiology, our findings might be relevant for designing treatment strategies. Non-invasive neuromodulation has been widely explored as a treatment alternative for migraine prevention (48). The visual cortex is a target of choice for such methods (49). If hyper-responsiveness in migraine were to be considered as resulting from of a network dysfunction rather than a primary local phenomenon, novel therapeutic opportunities may arise. Indeed, as shown in recent studies, dorsal fronto-parietal areas can causally modulate the activity of visual areas (35) and anodal transcranial direct current stimulation of the left temporal pole is able to normalize visual responses in migraine patients (50). Rather than targeting the primary visual cortex, which yielded equivocal results in therapeutic trials (48), non-invasive neurostimulation of other areas in the visual attention network could be more effective for the preventive treatment of migraine.

Our study has several limitations worth to mention. Although st-VEP analyses were fully automated, investigators performing VEP recordings were not blinded to the patients' diagnosis. In addition, the visual stimulus we employed is not suited to selectively stimulate the magnocellular or parvocellular visual pathways (51, 52), ventral or dorsal visual systems, or specifically evaluate attention. Moreover, the question remains open whether contralateral monocular (or a more physiological binocular) stimulation could have yielded different results; only future studies could provide a categorical answer to this question. In relation to electrode type and placement, superficial multichannel recordings would have provided much valuable information concerning spatial localization of visually-induced activity, and also, they would have allowed the implementation of sophisticated methods of artifact rejection to better reduce contamination by ocular movements. Concerning neuroimaging, because of technical issues scans from one participant could not be included in fMRI connectivity analyses (see methods section). With regards to the migraine status, we did not record patients during an attack, which made us unable to capture any possible cyclic changes in st-VEP responses, as well as to draw any firm conclusions concerning their pathophysiologic implication in headache crises. In what it respects to the participants, given that our migraine group was comprised only by migraine without aura patients, our results cannot be extrapolated to migraine with aura or its electrophysiological signature: cortical spreading depression. Finally, although likely reflecting the real-world situation, most female patients in both migraine and control groups were under hormonal contraception, which could affect cortical excitability.

To conclude, mean st-VEP amplitude is greater in episodic migraine patients compared to controls, and its positively correlated with gray matter volume in several functionally interconnected cerebral areas involved in visual processing, which mainly belong to the ventral attention network. Visual hypersensitivity in migraine seems to be a complex multi-regional process coupled to stimulus driven attention systems rather than an alteration restricted to the visual cortex.

## Availability of data and materials

Further data from the underlying research material can be obtained upon request to the corresponding author.

## Author contributions

ML contributed in the study design, data acquisition, data processing (focus on electrophysiology), statistical analyses, and wrote the first draft. KD contributed in the study design, data acquisition, data processing (focus on neuroimaging) and statistical analyses. GC, AM, JS, and DM contributed in the study design and revised the drafts of the manuscript. VP revised the drafts of the manuscript. All authors read and approved the final version.

### Conflict of interest statement

The authors declare that the research was conducted in the absence of any commercial or financial relationships that could be construed as a potential conflict of interest.

## Abbreviations

VBM: voxel-based morphometry MRI
HV: healthy volunteers
EM: episodic migraine without aura
ICHD: International Classification of Headache Disorders
st-VEP: Single trial visual evoked potentials
rTPJ: right temporo-parietal junction
ROI: region of interest
fc-MRI: resting state functional connectivity MRI.
